# Invasive Klebsiella pneumoniae Causing Concurrent Liver and Pulmonary Abscesses: Successful Management With Prolonged Oral Amoxicillin-Clavulanate

**DOI:** 10.7759/cureus.87412

**Published:** 2025-07-07

**Authors:** Mohammed Y Ishag, Abdu Latif Alsuleimani

**Affiliations:** 1 Internal Medicine, Nizwa Hospital, Nizwa, OMN; 2 Internal Medicine, Sudan Medical Specialization Board (SMSB), Khartoum, SDN; 3 Gastroenterology, Nizwa Hospital, Nizwa, OMN

**Keywords:** diabetes mellitus (dm), hypervirulent klebsiella pneumoniae (hvkp), invasive klebsiella pneumoniae liver abscess syndrome (ikplas), invasive klebsiella pneumoniae syndrome (ikps), outpatient parenteral antibiotic therapy (opat) program

## Abstract

We report a rare case of a 59-year-old male patient with poorly controlled diabetes mellitus who presented with concurrent hepatic and pulmonary abscesses. Imaging revealed a large septated hepatic lesion and multiple bilateral pulmonary cavities. Cultures from blood and drained hepatic pus confirmed *Klebsiella pneumoniae*. Despite the lack of genomic testing, the clinical course was consistent with invasive *Klebsiella pneumoniae* syndrome (IKPS). The patient was treated successfully with prolonged intravenous and oral amoxicillin-clavulanate over six months, along with percutaneous drainage of the hepatic abscess. Follow-up imaging demonstrated near-complete resolution of both liver and lung lesions. This case highlights the importance of early source control and extended culture-guided antibiotic therapy in managing severe invasive *Klebsiella* infections in immunocompromised patients.

## Introduction

*Klebsiella pneumoniae* is a gram-negative bacterium known to cause a wide spectrum of infections, including pneumonia, urinary tract infections, and liver abscesses [1]. However, simultaneous involvement of both the liver and lung is uncommon and often associated with hypervirulent strains [2]. Hypervirulent* Klebsiella pneumoniae* (hvKP) has emerged as a distinct pathotype capable of causing severe, community-acquired infections with metastatic spread, even in otherwise healthy individuals. These strains are characterized by increased virulence factors such as capsular serotypes K1 and K2 and are often associated with liver abscesses complicated by endophthalmitis, meningitis, or pulmonary involvement [3]. Here, we report a rare case of concurrent liver and pulmonary abscesses caused by *Klebsiella pneumoniae *that responded to a prolonged course of amoxicillin-clavulanate.

## Case presentation

A 59-year-old male patient, with a background of poorly controlled type 2 diabetes mellitus on insulin, a history of alcohol use (ceased), and past surgical resection of a complicated pancreatic pseudocyst 20 years prior, had received a short course of oral amoxicillin capsules for a presumed chest infection approximately one month before and presented with upper abdominal pain, associated with intermittent fever, unintentional weight loss, and loss of appetite. On examination, he appeared dehydrated with minimal jaundice and afebrile. Abdominal examination revealed mild tenderness in the right hypochondrium without hepatosplenomegaly or peripheral edema. Respiratory examination noted decreased air entry at the right lower lung field. Initial laboratory investigations are summarized in Table 1.

**Table 1:** Initial laboratory investigations Hb: hemoglobin; TWBCs: total white blood cells; PLT: platelet; ALT: alanine transaminase; AST: aspartate transaminase; ALP: alkaline phosphatase; HbA1c; hemoglobin A1c; CEA: carcinoembryonic antigen; AFP: alpha-fetoprotein; HBV: hepatitis B virus; HCV: hepatitis C virus; HIV: human immunodeficiency virus

Investigations	Results	Normal values
Hb	12	14-16.5 g/dL
TWBCs	18	4-11 x 10⁹/L
Neutrophils	16.3	2.5-8 x 10⁹
PLT	101	150-400 x 10⁹/L
CRP	336	<3 mg/L
Urea	8	2.5-7.8 mmol/L
Creatinine	138	44-97 µmol/L
Total bilirubin	26.3	5.1-17 µmol/L
ALT	228	10-55 IU/L
AST	413	10-40 IU/L
ALP	402	44-147 IU/L
Albumin	32	35-50 g/L
HbA1c	9.5%	3.5-6%
CEA	0.8	0-2.5 ng/ml
AFP	10	<40 ng/ml
HBV/HCV/HIV	Not detected	-
Stool culture	No growth	-

**Figure 1:**
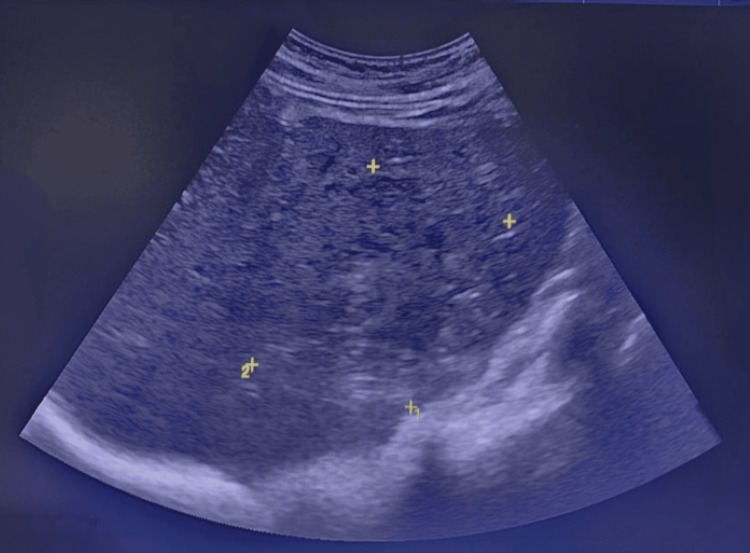
An abdominal ultrasound revealed a solid focal mass in the right hepatic lobe measuring 10 x 8.5 cm with mixed echotexture and cystic changes

**Figure 2:**
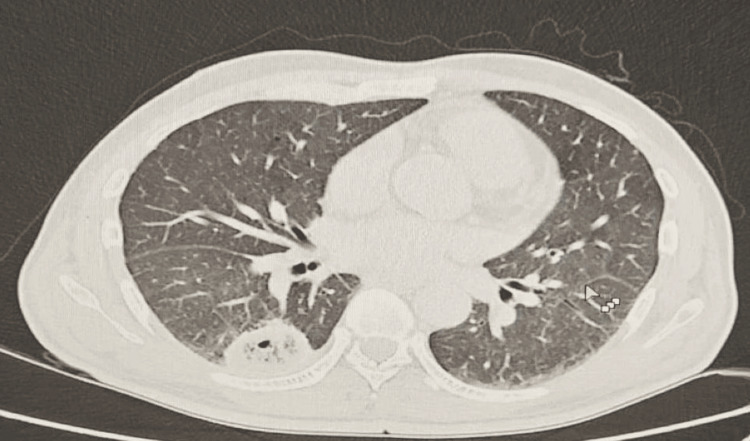
Contrast-enhanced CT chest showed multiple bilateral pulmonary cavitary lesions, largest in the right lower lobe measuring 29 x 44 x 33 mm

**Figure 3:**
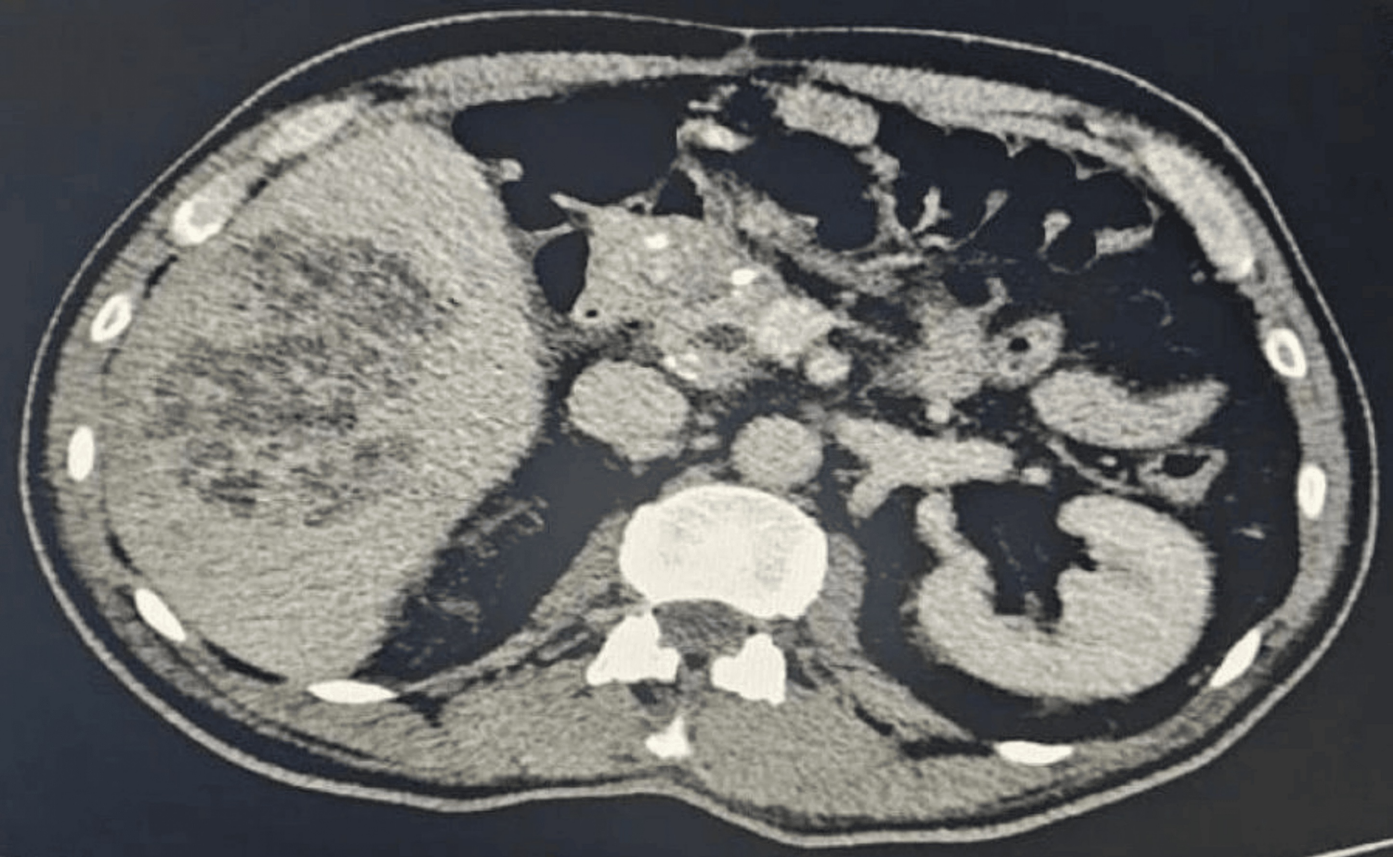
Contrast-enhanced CT showed a large septated mass in the right hepatic lobe (88 x 61 x 84 mm) and an adjacent mass (36 x 26 x 39 mm) with a bulky pancreatic head and dilated pancreatic duct (9 mm)

Abdominal ultrasound revealed a solid focal mass in the right hepatic lobe measuring 10 x 8.5 cm with mixed echotexture and cystic changes (Figure 1). Contrast-enhanced CT showed a large septated mass in the right hepatic lobe (88 x 61 x 84 mm) and an adjacent mass (36 x 26 x 39 mm), along with multiple bilateral pulmonary cavitary lesions (largest in the right lower lobe measuring 29 x 44 x 33 mm) (Figure 2). Additionally, a bulky pancreatic head with a dilated pancreatic duct (9 mm) was noted (Figure 3).

**Figure 4:**
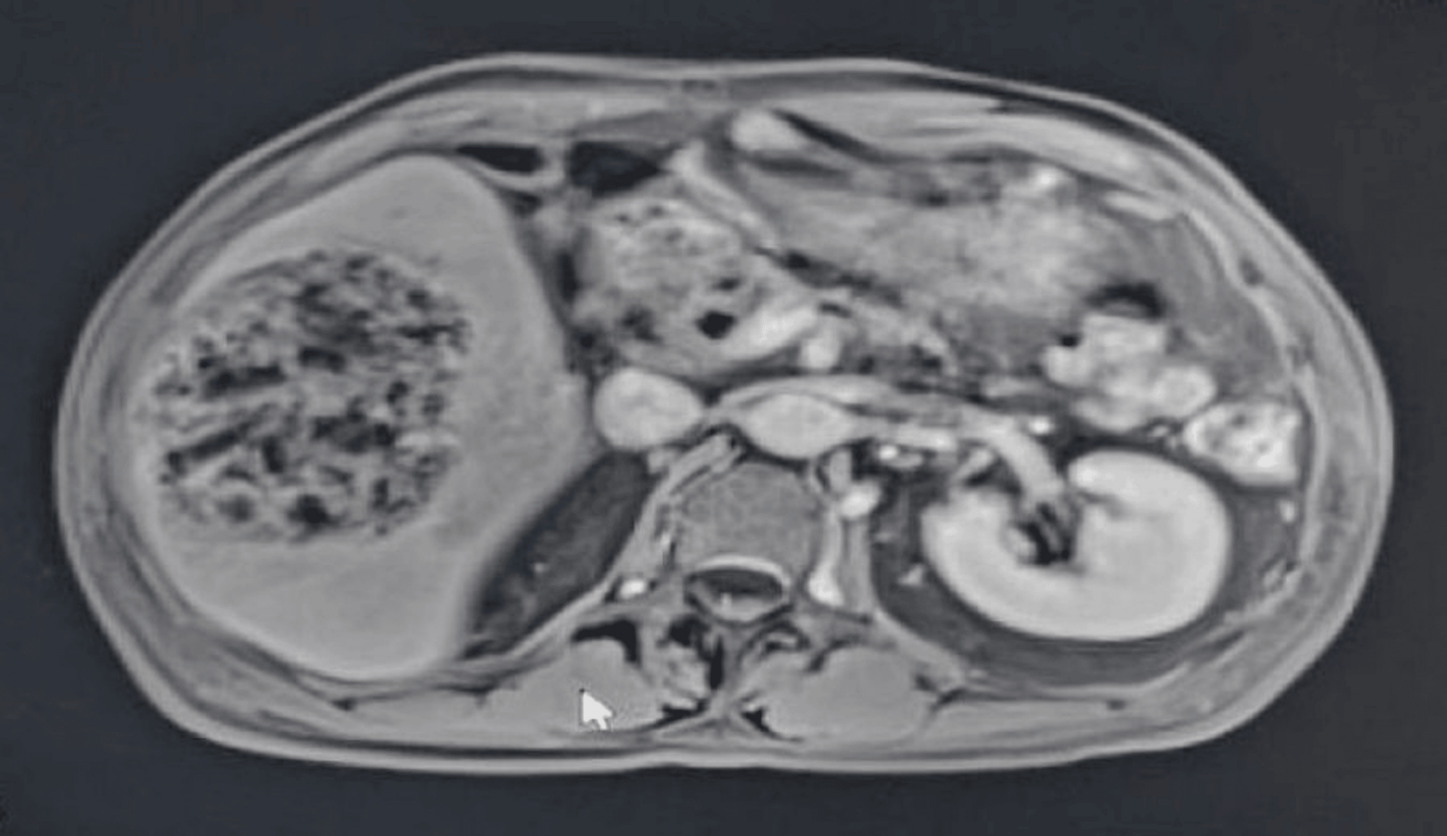
An MRI of the liver showed a large septated cystic hepatic lesion (9.4 x 7.3 x 9.4 cm)

The patient was initiated on intravenous piperacillin-tazobactam and metronidazole, along with intravenous fluids and strict diabetic control. Blood cultures grew *Klebsiella pneumoniae*, sensitive to amoxicillin-clavulanate, cefuroxime, ciprofloxacin, gentamicin, and trimethoprim-sulfamethoxazole. An MRI of the liver showed a large septated cystic hepatic lesion (9.4 x 7.3 x 9.4 cm) and thrombosis of a segment of the right hepatic vein (Figure 4). Endoscopic ultrasound revealed a hypoechoic pancreatic head lesion with calcifications, but the biopsy was deferred due to anticoagulation.

One week after admission, percutaneous drainage of the hepatic abscess was performed by the interventional radiology team. Pus culture also grew *Klebsiella pneumoniae* with a similar sensitivity profile, with resistance to ampicillin. Despite the unavailability of genomic testing, the clinical pattern was highly suggestive of invasive *Klebsiella pneumoniae* syndrome (IKPS), and management was tailored accordingly.

After 14 days of piperacillin-tazobactam, the patient was de-escalated to intravenous amoxicillin-clavulanate (1.2 g every 8 hours). Serial ultrasounds demonstrated a reduction in hepatic abscess size (down to 5.5 x 6.4 cm), and the pigtail catheter was removed three weeks after insertion due to cessation of drainage. The patient became afebrile, symptoms resolved, and inflammatory markers improved significantly.

He was discharged on prolonged oral amoxicillin-clavulanate (1.2 g every 8 hours) through an outpatient parenteral antibiotic therapy (OPAT) program. He also received oral rivaroxaban due to hepatic vein thrombosis. An infectious disease (ID) consultant was actively involved and remained a core member of the multidisciplinary team (MDT) throughout the patient's management.

**Figure 5:**
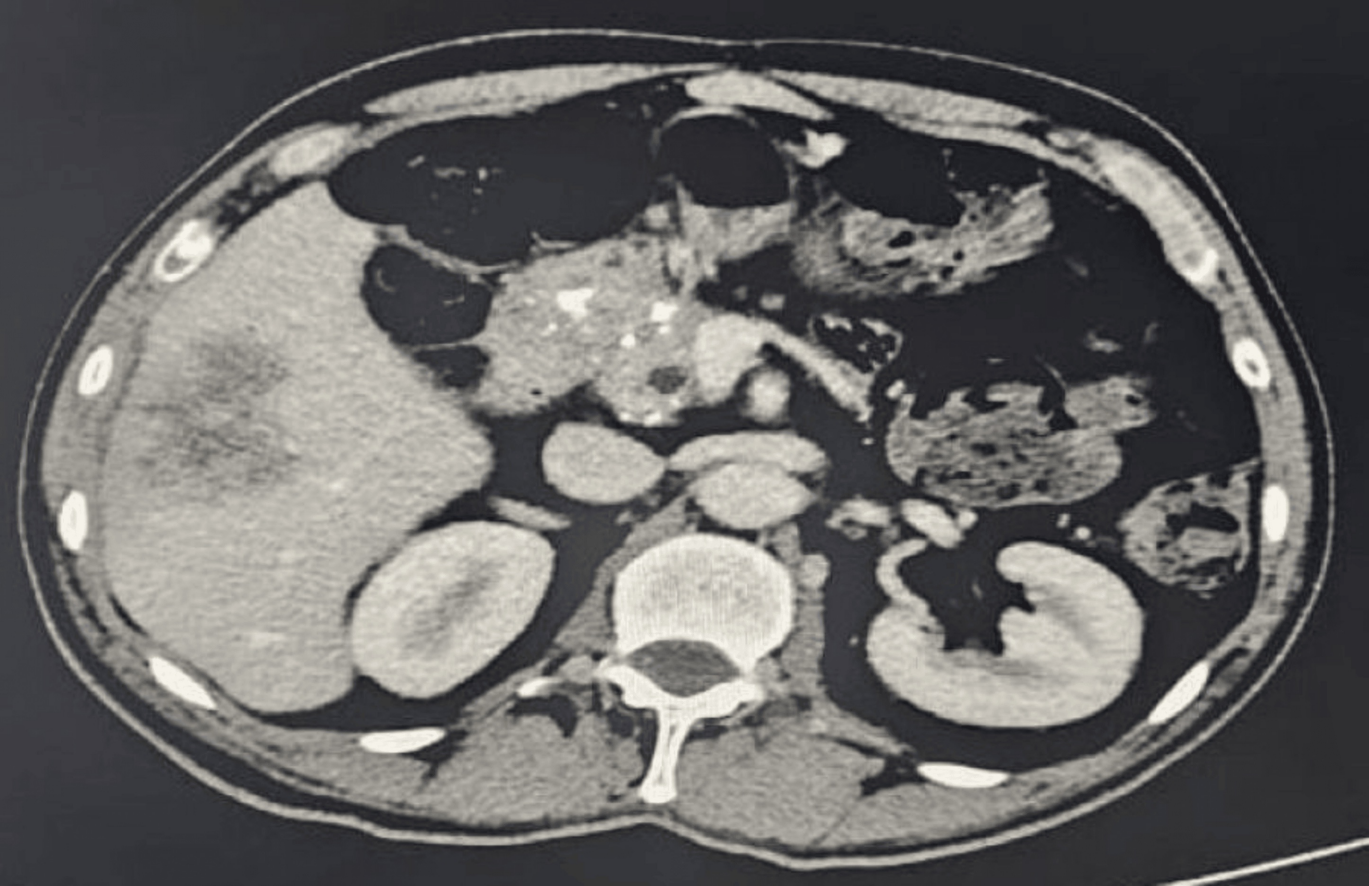
Contrast-enhanced CT after four months of treatment showed a reduction of the hepatic lesion measuring about 28 x 20 x 15 mm

**Figure 6:**
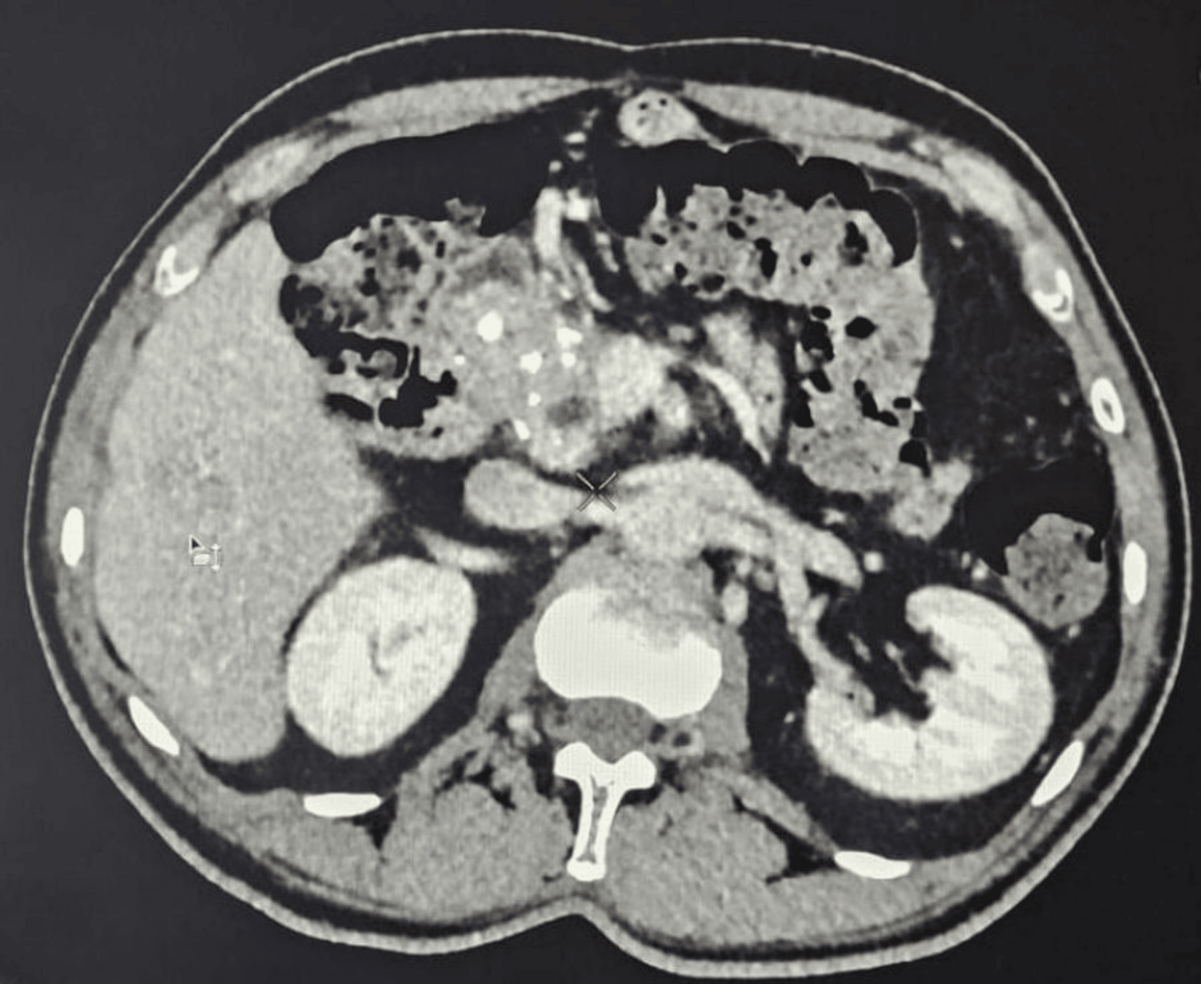
Contrast-enhanced CT showed the hepatic lesion with marked reduction in size and density appearing as subtle irregular faint hypodensity

**Figure 7:**
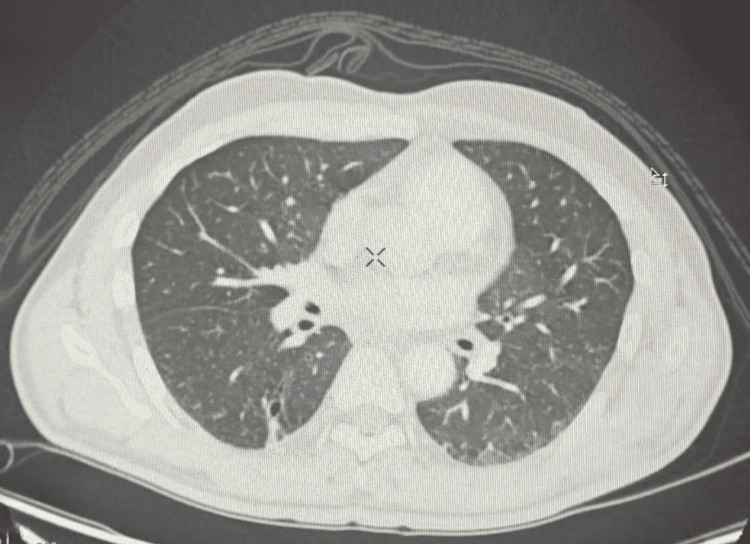
CT chest demonstrated the right lower lobe lesion, appearing as air cyst/thin-walled cavity measuring 5 x 7 x 13 mm surrounding with linear atelectatic bands

At four months, follow-up CT showed a reduced right hepatic lesion (28 x 20 x 15 mm) and a smaller pulmonary cavity (Figure 5). Rivaroxaban was discontinued, and antibiotics were continued orally. After completing a six-month antibiotic course, a final CT revealed a residual thin-walled cavity in the right lower lobe and near-complete resolution of the hepatic lesion (Figures 6, 7). With normalized inflammatory markers and clinical improvement, antibiotics were discontinued, and the patient remains under surveillance.

## Discussion

hvKP has emerged globally as an important pathogen capable of causing highly invasive infections. Originally described in the 1980s in Taiwan, hvKP was identified as the causative agent of community-acquired liver abscesses with metastatic complications such as endophthalmitis and meningitis, even in otherwise healthy individuals [3,4]. Over time, the syndrome has been reported across Europe, North America, and Australia, although the Asia-Pacific region remains the predominant area of incidence [5]. Cases involving simultaneous liver and pulmonary abscesses remain particularly rare and underreported, making this case an important addition to the growing literature on hvKP.

The hypermucoviscosity phenotype of hvKP is attributed to the overproduction of capsular polysaccharides and virulence genes such as rmpA and capsular serotypes K1 and K2, which confer the organism’s resistance to phagocytosis. This phenotype is often identified by the "string test," wherein a viscous string >5 mm is produced from bacterial colonies [4,6].

Diabetes mellitus (DM) is one of the most significant risk factors for *Klebsiella pneumoniae *liver abscess (KLA), with prevalence ranging from 29% to 44% in reported cases [5,7,8]. Poor glycaemic control impairs neutrophil function and enhances the pathogenicity of K1/K2 strains, contributing to systemic dissemination [5]. Interestingly, studies have shown that prior exposure to aminopenicillins, particularly ampicillin or amoxicillin, is independently associated with an increased risk of KLA, possibly due to the disruption of intestinal microbiota and selective overgrowth of hypervirulent strains capable of translocation to the liver [9].

Invasive *Klebsiella pneumoniae* liver abscess syndrome (IKPLAS) is defined as KLA with one or more extrahepatic complications, the most common being endophthalmitis and pulmonary abscesses [8]. In our patient, the presence of both a large hepatic abscess and multiple pulmonary cavitary lesions fulfilled the diagnostic criteria for IKPLAS, even though confirmatory string testing was unavailable.

Thrombophlebitis involving hepatic or portal veins is a recognized complication of KLA, although the need for anticoagulation remains controversial [10]. One study reported thrombophlebitis in 31% of KLA patients, mostly involving the hepatic vein, with no clear benefit established for anticoagulation [11]. However, in select cases, anticoagulation may reduce the risk of septic embolization. Our patient had segmental hepatic vein thrombosis and was treated with rivaroxaban for three months, with good clinical and radiological outcomes.

Imaging plays a critical role in the diagnosis and management of KLA. CT characteristics suggestive of KLA include solitary, unilobar, solid, multiloculated abscesses with frequent venous thrombosis [12]. These features can complicate percutaneous drainage, which remains essential for source control in larger lesions (>5 cm). Our patient underwent image-guided pigtail drainage after stabilization, resulting in progressive resolution of the abscess.

hvKP is typically susceptible to many antibiotics except ampicillin, but there are growing concerns about resistance [3]. A retrospective study found that extended-spectrum cephalosporins had better outcomes than cefazolin in KLA treatment [13]. However, prolonged oral antibiotic therapy can also be effective, particularly when guided by sensitivity testing and combined with OPAT. Our case demonstrated excellent response to amoxicillin-clavulanate, both intravenously and orally, in line with a study from Singapore, which found that oral step-down therapy after five days of intravenous antibiotics was non-inferior to continued IV therapy for 12 weeks [14,15].

In some patients, especially those with persistent radiologic abnormalities or impaired immunity, a prolonged course of antibiotics may be required. Abscess cavities may persist radiographically despite clinical resolution, and should be closely monitored [15]. Our patient received a six-month course of antibiotics with regular imaging follow-up, driven by persistent radiologic findings and suboptimal glycaemic control. He eventually achieved complete clinical and radiologic resolution, reinforcing the importance of individualized treatment duration. This case also emphasizes a key clinical lesson: successful management of invasive hvKP infections is possible through a multidisciplinary approach, culture-guided prolonged oral therapy, and strict glycemic monitoring, even in high-risk patients.

## Conclusions

IKPS should be considered in diabetic patients presenting with concurrent hepatic and pulmonary abscesses. Prompt recognition, source control through drainage, and prolonged, targeted antibiotic therapy can lead to excellent clinical outcomes even in the absence of genomic confirmation. This case demonstrates the efficacy of extended treatment with amoxicillin-clavulanate and highlights the importance of individualized treatment strategies and follow-up in managing rare but severe manifestations of IKPS.

## References

[1] Monteiro AS, Silva MO, Galvão VS (2025). High proportions of multidrug-resistant Klebsiella pneumoniae isolates in community-acquired infections, Brazil. Sci Rep.

[2] Doshi S, Forbes JD, Mubareka S, Andany N (2022). Disseminated hypervirulent Klebsiella pneumoniae causing endophthalmitis, and lung and liver abscesses. CMAJ.

[3] Lee CR, Lee JH, Park KS (2017). Antimicrobial resistance of hypervirulent Klebsiella pneumoniae: epidemiology, hypervirulence determinants, and resistance mechanisms. Front Cell Infect Microbiol.

[4] Paczosa MK, Mecsas J (2016). Klebsiella pneumoniae: going on the offense with a strong defense. Microbiol Mol Biol Rev.

[5] Cheong XP, Lim LM, Chang CY (2024). Invasive Klebsiella pneumoniae syndrome: a case report from Malaysia. Cureus.

[6] Qian Y, Wong CC, Lai S (2016). A retrospective study of pyogenic liver abscess focusing on Klebsiella pneumoniae as a primary pathogen in China from 1994 to 2015. Sci Rep.

[7] Tian LT, Yao K, Zhang XY (2012). Liver abscesses in adult patients with and without diabetes mellitus: an analysis of the clinical characteristics, features of the causative pathogens, outcomes and predictors of fatality: a report based on a large population, retrospective study in China. Clin Microbiol Infect.

[8] Gu L, Wang Y, Wang H, Xu D (2025). Analysis of clinical and microbiological characteristics of invasive Klebsiella pneumoniae liver abscess syndrome. BMC Infect Dis.

[9] Lin YT, Liu CJ, Yeh YC, Chen TJ, Fung CP (2013). Ampicillin and amoxicillin use and the risk of Klebsiella pneumoniae liver abscess in Taiwan. J Infect Dis.

[10] Fadeyi O, Sovyanhadi Z, Gupta L, Dang V, Katamreddy Y, Hakim A (2023). Thrombosis, septic emboli, and multiple abscesses triggered by Klebsiella pneumoniae: a case report and literature review. J Investig Med High Impact Case Rep.

[11] Molton JS, Chee YL, Hennedige TP, Venkatesh SK, Archuleta S (2015). Impact of regional vein thrombosis in patients with Klebsiella pneumoniae liver abscess. PLoS One.

[12] Alsaif HS, Venkatesh SK, Chan DS, Archuleta S (2011). CT appearance of pyogenic liver abscesses caused by Klebsiella pneumoniae. Radiology.

[13] Cheng HP, Siu LK, Chang FY (2003). Extended-spectrum cephalosporin compared to cefazolin for treatment of Klebsiella pneumoniae-caused liver abscess. Antimicrob Agents Chemother.

[14] Molton JS, Chan M, Kalimuddin S (2020). Oral vs intravenous antibiotics for patients with Klebsiella pneumoniae liver abscess: a randomized, controlled noninferiority study. Clin Infect Dis.

[15] Jun JB (2018). Klebsiella pneumoniae liver abscess. Infect Chemother.

